# Emerging trends and knowledge networks in pan-cancer sorafenib resistance: a 20-year bibliometric investigation

**DOI:** 10.3389/fphar.2025.1581820

**Published:** 2025-07-23

**Authors:** Qiong Zhou, Rui Wang

**Affiliations:** Laboratory of Medical Oncology, Jinling Hospital, Affiliated Hospital of Medical School, Nanjing University, Nanjing, China

**Keywords:** sorafenib resistance, bibliometrics, pan-cancer, knowledge network, targeted therapy

## Abstract

**Background:**

Sorafenib, a multi-kinase inhibitor, is a key therapeutic agent in the treatment of advanced hepatocellular carcinoma (HCC), metastatic renal cell carcinoma (RCC), and radioactive iodine-refractory differentiated thyroid cancer (DTC). However, its clinical efficacy is frequently hampered by the rising prevalence of sorafenib resistance, particularly in HCC. This reality underscores the urgent need for a comprehensive pan-cancer investigation to elucidate the underlying mechanisms of resistance.

**Methods:**

We employed a systematic bibliometric approach utilizing the Web of Science Core Collection to conduct a structured literature search. Performance analysis and visualization were conducted using VOSviewer and CiteSpace. A triphasic screening protocol was implemented to identify publications focused on sorafenib resistance, covering a period from 2006 to 2025.

**Results:**

Our analysis identified 1,484 eligible publications, with a peak of 194 articles published in 2022. The majority of research (79.48%) centered on HCC, with significant contributions from institutions in China and the United States. Co-authorship and keyword network analyses revealed a robust interdisciplinary collaboration landscape. Key themes emerged, including dysregulation of drug transporters and clearance mechanisms, metabolic reprogramming, programmed cell death, interactions within the tumor microenvironment, and epigenetic regulatory mechanisms, highlighting critical areas contributing to resistance.

**Conclusion:**

This study highlights the current research landscape concerning sorafenib resistance, facilitating the identification of emerging trends and research gaps. Future research priorities should include biomarker-driven clinical trials, the development of nanoparticle delivery systems, and the clinical translation of combination therapy strategies. Additionally, the integration of deep learning algorithms in the context of big data has the potential to enhance our understanding of resistance mechanisms *in silico*, ultimately aiming to overcome resistance challenges and improve patient survival outcomes.

## Graphical Abstract

**Figure:**
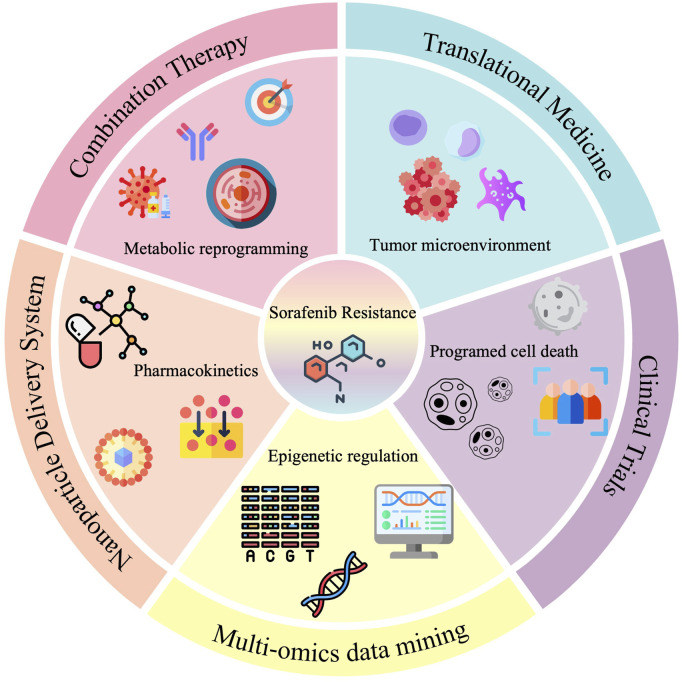
*Knowledge Framework of Sorafenib Resistance*: *Mechanisms and Future Directions* This three-layered conceptual map synthesizes 20 years of research on sorafenib resistance across cancers: Core (Central Circle): *Sorafenib Resistanc*e — The focal theme of this bibliometric analysis, reflecting its pan-cancer clinical challenge; Intermediate Layer (Mechanisms): Six key resistance drivers identified through literature mining; Outer Layer (Solutions): Emerging strategies to overcome resistance.

## Introduction

Sorafenib, a multi-kinase inhibitor targeting pathways involved in proliferation and angiogenesis, with specific targets including Raf1, B-Raf, VEGFR1-3, PDGFR-β, c-Kit, and FLT-3, has been a cornerstone in the treatment of metastatic renal cell carcinoma (RCC), advanced hepatocellular carcinoma (HCC), and radioactive iodine-refractory differentiated thyroid cancer (DTC) (Wilhelm et al., 2004; Escudier et al., 2007; Llovet et al., 2008; Brose et al., 2014). Initial clinical trials demonstrated median survival benefits of 2.7 months in HCC (SHARP trial) and RCC (TARGET trial) (Escudier et al., 2007; Llovet et al., 2008). However, in advanced HCC, approximately 60%–70% of patients exhibit innate resistance (primary progression), while 30%–40% develop acquired resistance within 6 months through adaptive mechanisms (Cheng et al., 2009; Gauthier and Ho, 2013). Although survival rates tend to be more favorable in renal and thyroid cancers, the overall therapeutic efficacy of sorafenib remains limited, particularly in HCC (E et al., 2019; Huang et al., 2022; Jiang et al., 2025).

The current understanding of resistance mechanisms poses a fundamental barrier to sorafenib’s clinical efficacy (Zhu et al., 2017). While numerous studies have characterized tumor-specific resistance pathways, a systematic pan-cancer analysis remains critically underexplored (Tang et al., 2020; He et al., 2021; Holm et al., 2022). Comparative pan-cancer investigations provide a powerful framework for identifying both evolutionarily conserved resistance mechanisms and context-dependent variations across malignancies. This paradigm not only facilitates the development of novel broad-spectrum therapeutic strategies but also enables biomarker-based patient stratification.

Bibliometric analysis has emerged as a powerful tool to map scientific landscapes, identifying knowledge clusters, collaborative networks, and emerging trends (Jo and shi, 2014; Chen, 2006). Previous studies have applied this methodology to oncology topics such as immunotherapy and precision medicine, yet no comprehensive bibliometric evaluation exists for sorafenib resistance across cancers (Ji et al., 2024; Li et al., 2023a; Meng et al., 2024). This gap hinders the identification of cross-disciplinary insights and translational opportunities.

Our study embarks on a 20-year bibliometric analysis spanning from 2006 to 2025 with the following objectives (Wilhelm et al., 2004): to quantify global research output trends related to sorafenib resistance (Escudier et al., 2007), to map interdisciplinary collaborations and institutional contributions in this domain, and (Llovet et al., 2008) to identify underexplored mechanisms via keyword co-occurrence and burst detection analyses.

## Materials and methods

**FIGURE 1:**
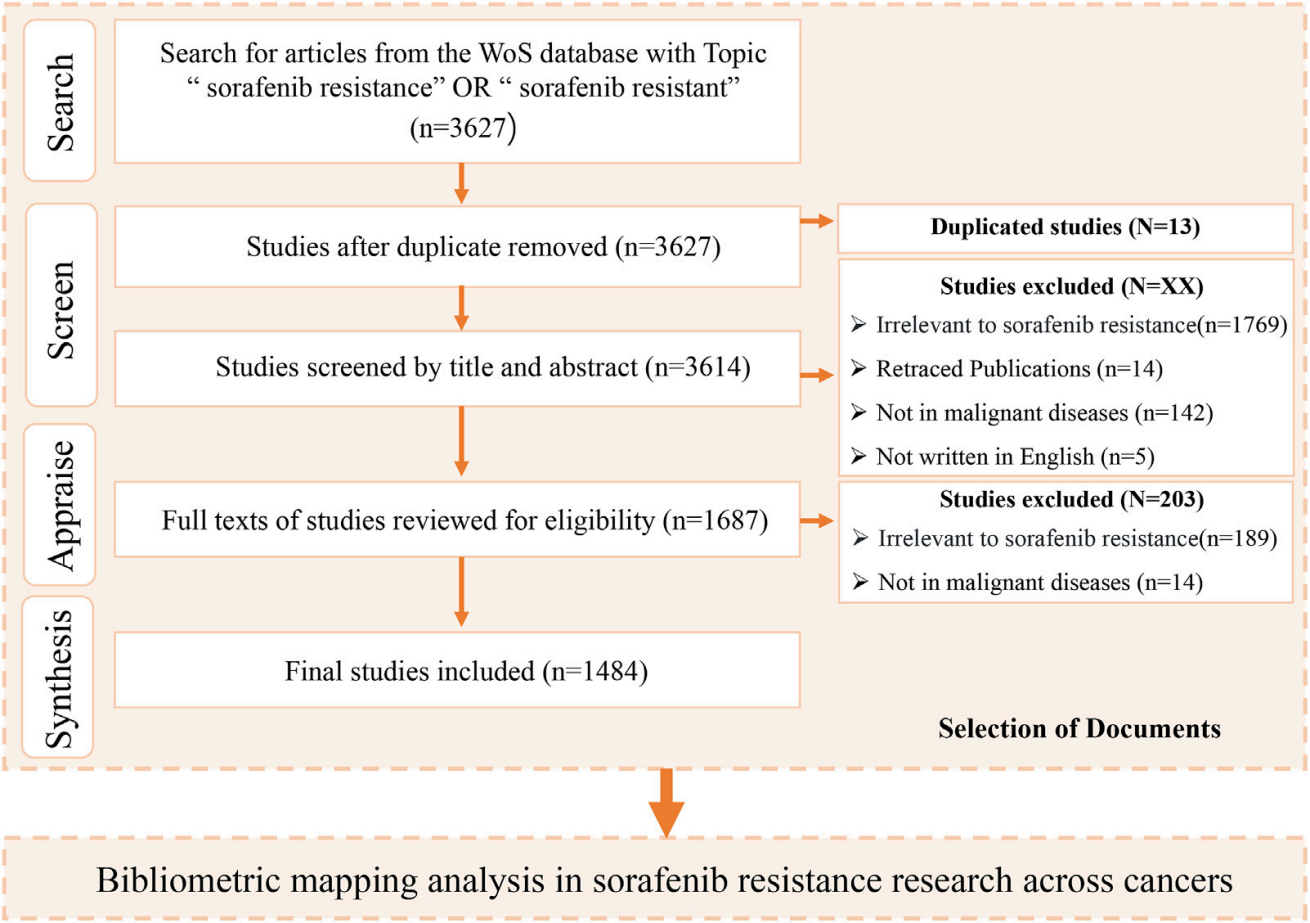
Prisma selection workflow diagram.

This investigation adopted a systematic bibliometric framework to elucidate sorafenib resistance mechanisms across heterogeneous tumor types, integrating quantitative performance metrics with qualitative network analytics. Data acquisition was conducted through the Web of Science Core Collection database, employing the SCI-EXPANDED and SSCI indices to optimize retrieval of high-impact publications. A structured search strategy {TS= [(“sorafenib resistance”) OR (“sorafenib resistant”)]} was implemented with temporal parameters spanning records published through 1 January 2025, filtered to include only original research articles and review articles. Post-retrieval validation involved a triphasic screening protocol: (1) title-level relevance assessment, (2) abstract-based thematic verification, and (3) full-text content analysis for mechanistic focus, with the detailed screening workflow schematized in Figure 1.

Bibliometric processing utilized VOSviewer (Version 1.6.18) and CiteSpace (Version 6.4.R1), two validated platforms for scient metric exploration (Pan et al., 2018). VOSviewer generated co-authorship networks and keyword co-occurrence matrices through its optimized clustering algorithms, effectively delineating interdisciplinary research consortia (Van Eck and Waltman, 2010). CiteSpace employed temporal slicing (1-year intervals) and Kleinberg’s burst detection to map citation trajectory evolution and emerging thematic frontiers (Chen, 2017).

## Results

### Literature overview on sorafenib resistance

Following a standardized data curation process, this study systematically analyzed 1,484 eligible publications, comprising 1,314 original research articles and 170 review articles. These works were contributed by 9,918 researchers affiliated with 1,666 institutions across 47 countries and published in 433 peer-reviewed journals. Cumulatively, the included publications received 68,405 citations (46.10 citations per article) as of the retrieval cutoff date (1 January 2006–1 January 2025), with an annual citation rate of 3,420. The H-index of 109 reflects the substantial scholarly impact of this corpus, integrating both productivity and citation influence.

**FIGURE 2:**
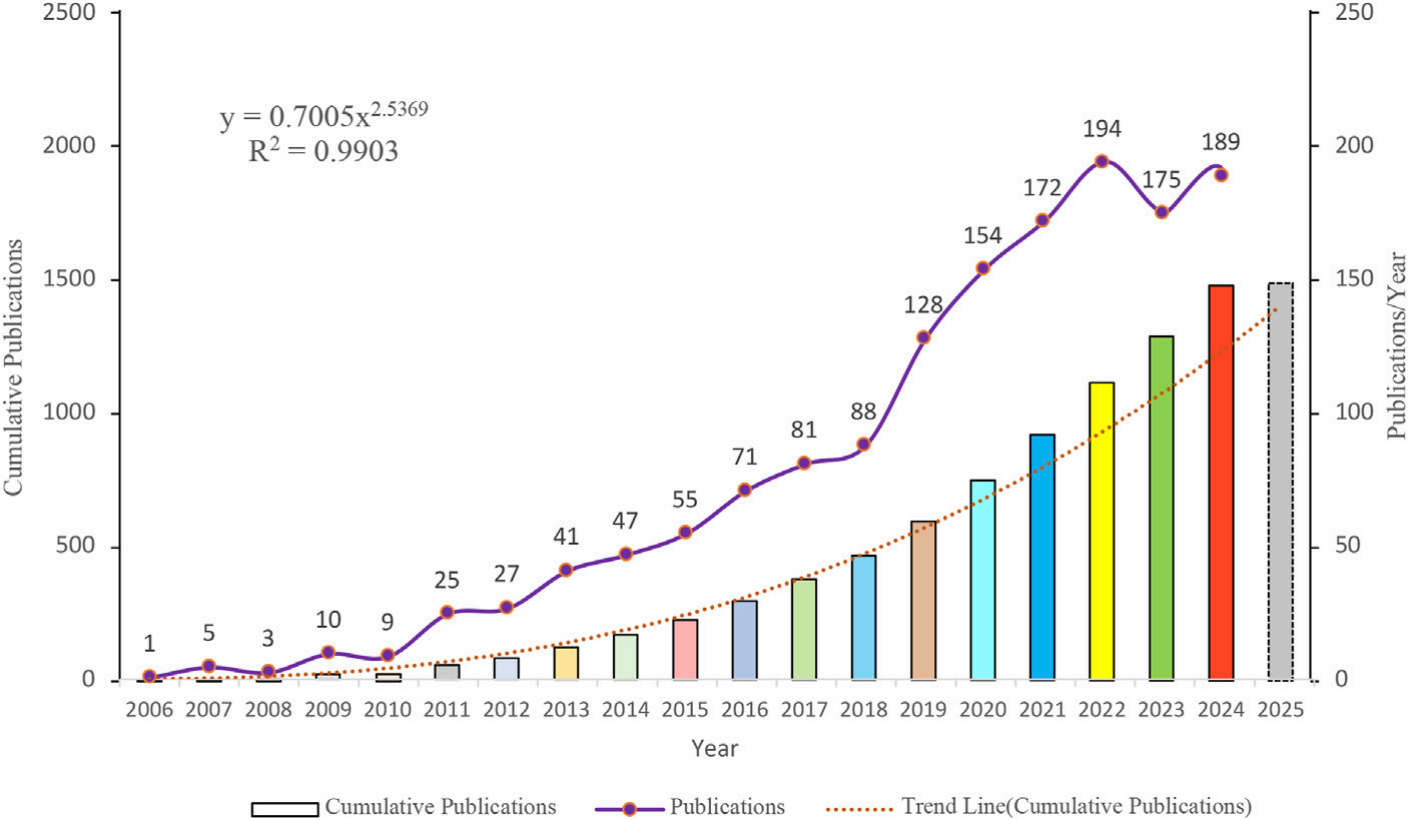
Temporal trends in article publications.

Temporal analysis revealed dynamic publication patterns in sorafenib resistance research (Figure 2). Annual output exhibited a pronounced growth trajectory, peaking at 194 publications in 2022. Despite a modest decline in subsequent years, productivity remained robust (≥175 articles/year). Cumulative publication growth demonstrated strong adherence to a power-law distribution (*R* ^2^ = 0.9903), indicative of sustained domain expansion. This model projects continued incremental increases in scholarly output, suggesting persistent research momentum in this therapeutic challenge.

### Analysis of principal research journals

Original research on sorafenib resistance has garnered escalating attention in high-impact oncology journals, positioning this topic as a priority in cancer therapeutics. Regional journal distribution patterns (Table 1) revealed alignment with Bradford’s Law of Scattering: the tripartite division exhibited a 1:3:3^2^ ratio in journal numbers per zone, with comparable article counts across zones (2006–2025), confirming the expected dispersion of scholarly output.

**TABLE 1:** Distribution of publications and journals in different zones.

Zone	Documents	Number of publications	Number of journals
First Zone	≥11	510	30
Second Zone	3–11	492	108
Third Zone	1–2	482	295

**FIGURE 3:**
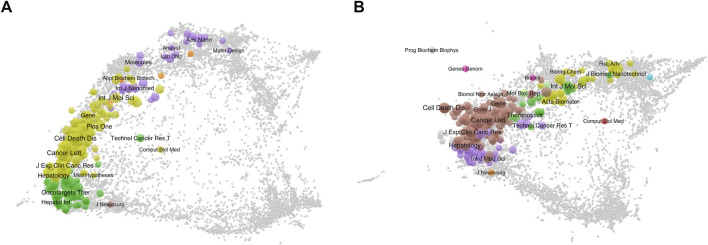
Overview of dual-map overlay. **(A)** Citing journals and **(B)** cited journals.

Figures 3A,B present a dual overlay analysis of citing and cited literature in the context of sorafenib resistance in cancer research. This visualization highlights the relationships and citation dynamics between various research areas and journals. The size and color of the nodes represent the citation frequency and categorization of different literature sources. The concept of dual overlay analysis serves as a powerful tool for understanding how specific research themes are interconnected within the academic landscape (Chen and Leydesdorff, 2014). By juxtaposing citing and cited literature, researchers can identify influential works that have shaped the discourse surrounding sorafenib resistance, as well as uncover emerging trends and potential gaps in the literature.

**FIGURE 4:**
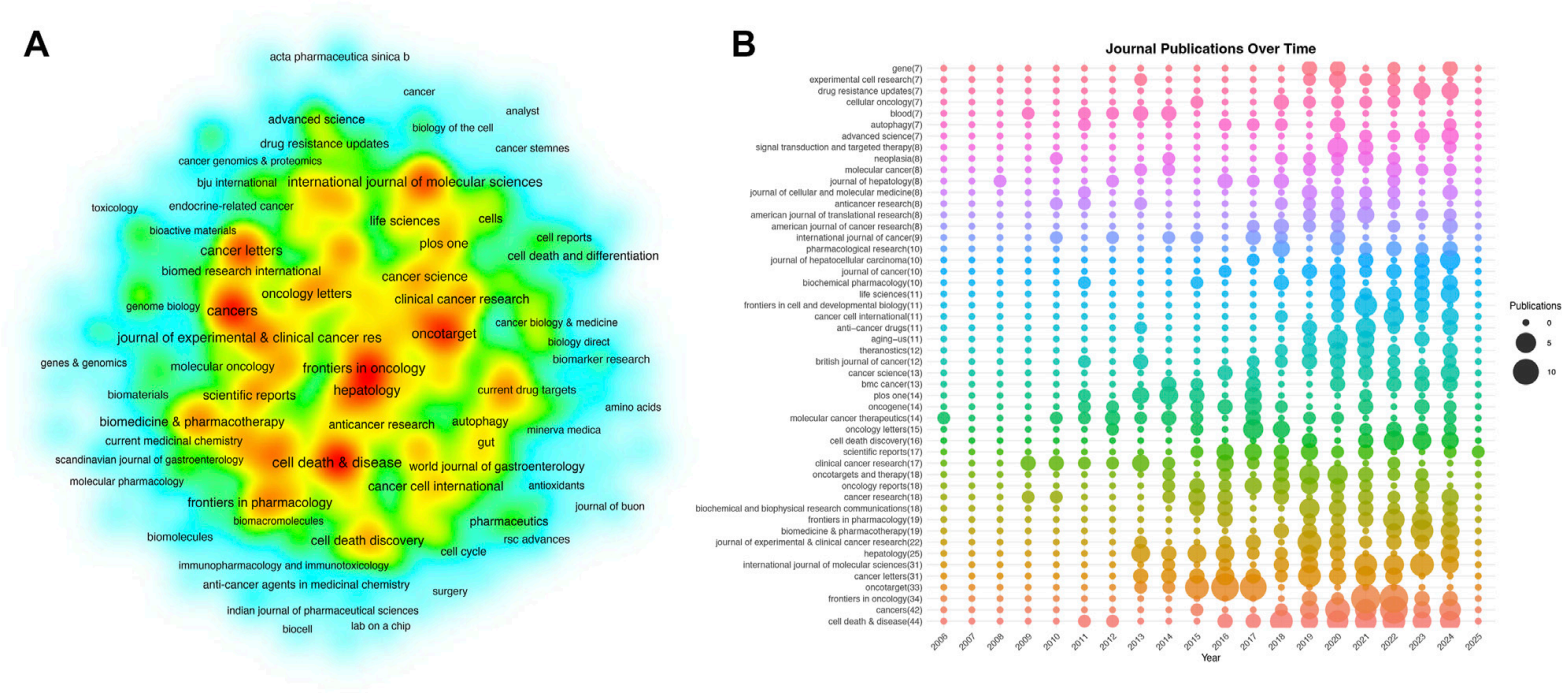
Main research journals analysis. **(A)** Density map of journal distribution. **(B)** Top 50 journals in terms of publications.

A density network map (Figure 4A) illustrates the global spread of journals publishing sorafenib resistance studies. The ranked list of top 50 journals (Figure 4B) further clarifies contributions, with 60% (30/50) originating from Bradford’s first zone (core journals). Cell Death & Disease led in publication volume (44 articles, 3.0% of total output), followed by Cancers (42 articles) and Frontiers in Oncology (34 articles). However, citation metrics revealed distinct leaders: Hepatology (4,269 total citations; 170.76 per article), Cell Death & Disease (2,135 citations; 48.52 per article), and Cancer Letters (1,977 citations; 63.77 per article) dominated citation impact, highlighting their pivotal roles in disseminating influential findings. The absence of a single dominant journal (e.g., Cell Death & Disease contributing only 3.0% of publications) underscores the field’s decentralized scholarly landscape. These findings not only identify high-impact publication platforms for researchers but also quantify the heterogenous distribution of authority within sorafenib resistance literature.

### Countries and Global Collaboration Patterns

**FIGURE 5:**
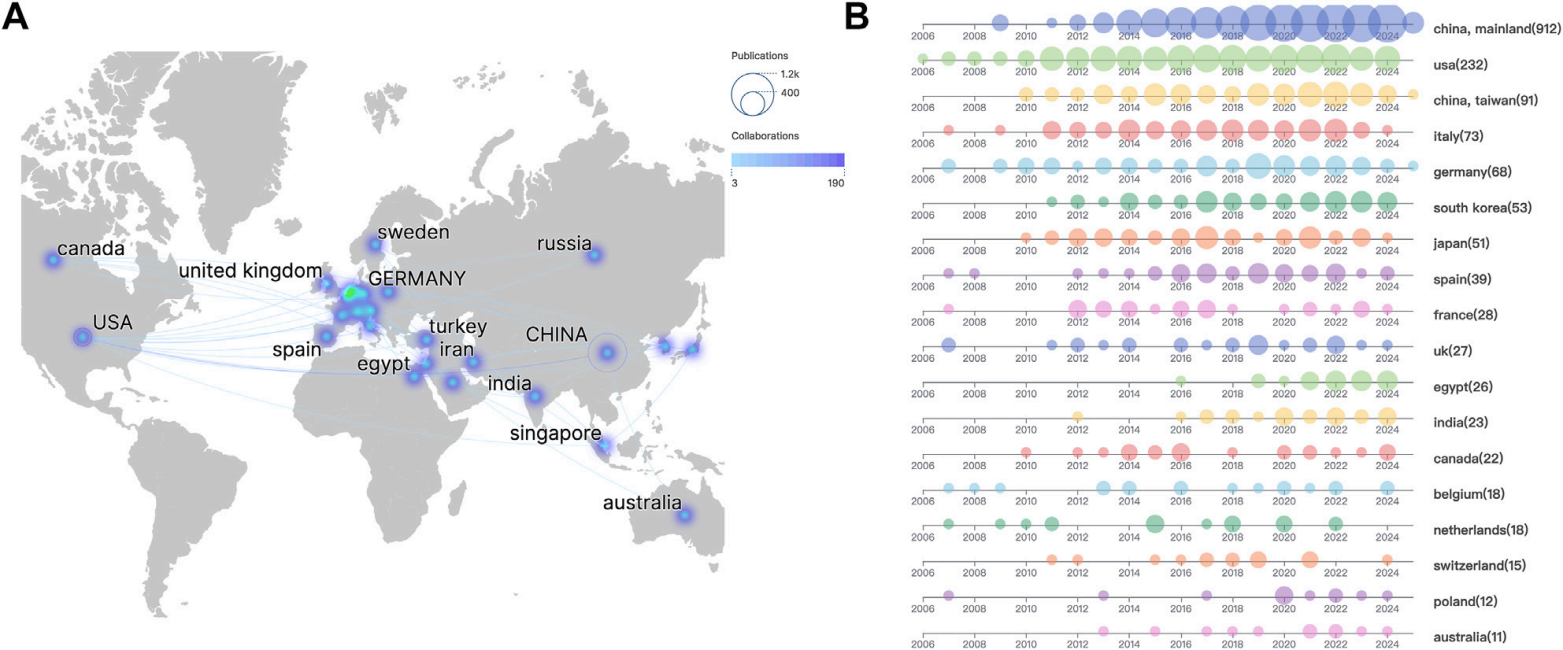
Global collaboration patterns in sorafenib resistance research **(A)** geographical disparities and collaboration networks. **(B)** Annual publication trends in high-output countries.

Bibliometric analysis revealed distinct geographical disparities in research output and collaboration networks (Figure 5A). China and the United States dominated total publications, collectively producing over twice the volume of other nations. The U.S. maintained strongest collaborative links with China, the United Kingdom, and Germany, as evidenced by darker interconnection lines, while partnerships with other countries exhibited lower connectivity density. Temporal analysis of annual publications across high-output countries (>10 publications) demonstrated dynamic patterns (Figure 5B). China (including Mainland and Taiwan region) showed continuous growth since 2010, contrasting with stable U.S. productivity. Post-2016, all 17 analyzed countries sustained consistent publication output. Notably, developing economies constituted only 17.65% of contributing nations (3/17: China, Egypt, India), reflecting concentrated research activity in developed countries.

Citation analysis uncovered divergent impact profiles. Despite China and the U.S. leading in publication quantity, Poland achieved the highest mean citations per article (333.00), followed by the United Kingdom (263.67) and France (247.11), indicating superior academic influence within European research consortia (Table 2).

**TABLE 2:** Research output and impact metrics of top contributing countries.

Rank	Country	Documents	Citations	Average citation	Total link strength
1	China	1003	36,861	36.75	6,027
2	United States	232	23,824	102.69	2,972
3	Italy	73	4,914	67.32	1034
4	Germany	68	7,298	107.32	971
5	South Korea	53	2,714	51.21	411
6	Japan	51	1915	37.55	533
7	Spain	39	3,969	101.77	957
8	France	28	6,919	247.11	630
9	United Kingdom	27	7,119	263.67	489
10	Egypt	26	482	18.54	230
11	India	23	461	20.04	264
12	Canada	22	1783	81.05	499
13	Belgium	18	1022	56.78	334
14	Netherlands	18	2,891	160.61	265
15	Switzerland	15	1262	84.13	204
16	Poland	12	3,996	333.00	171
17	Australia	11	289	26.27	221

### Institutions and authors analysis

**FIGURE 6:**
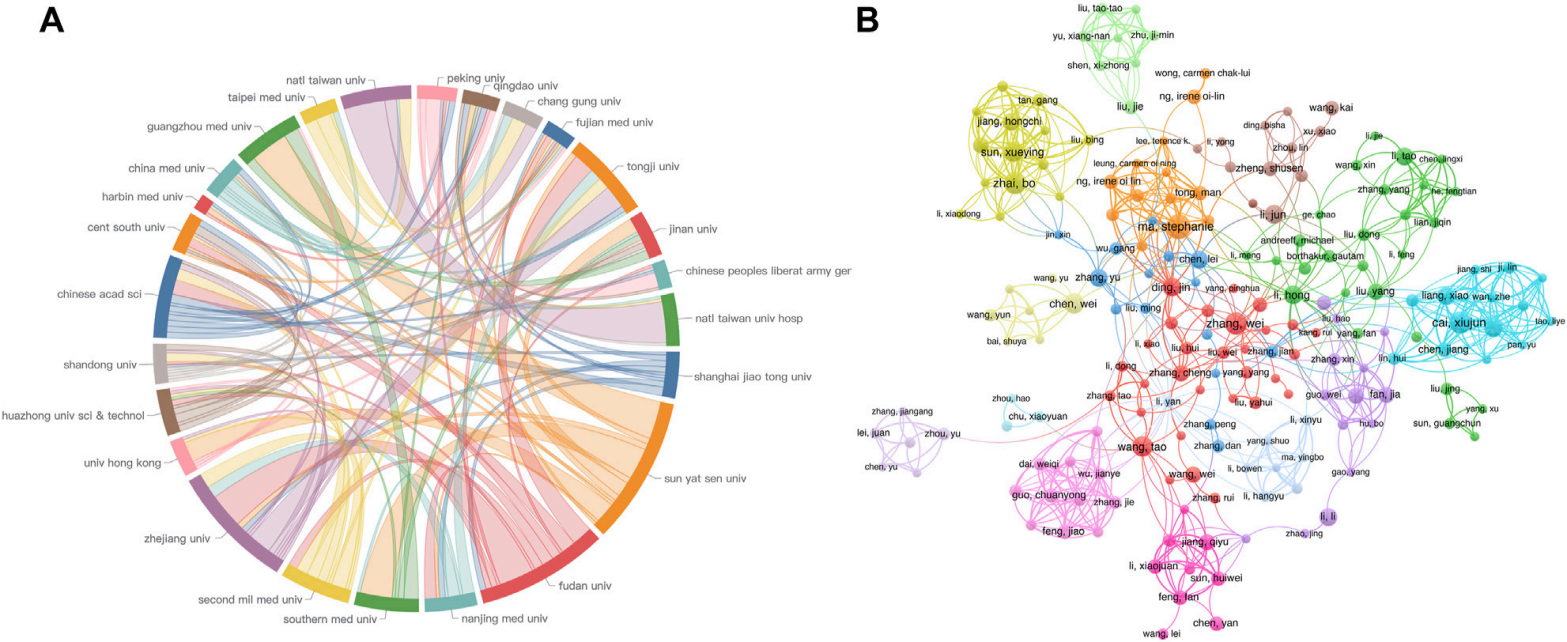
Collaboration networks of **(A)** institution and **(B)** authors.

To evaluate the research capabilities and scientific impact of institutions and authors in the field of sorafenib resistance studies, we performed a systematic analysis of publication volumes and corresponding citation counts. Figures 6A,B depict the publication numbers and collaboration networks among institutions and core authors, respectively. Figure 6A is a chord diagram depicting institutional collaborations, where the size of each sector indicates publication engagement, and thicker connecting lines represent more frequent interactions. Prominently, Zhejiang University, Sun Yat-sen University, and Fudan University emerge as the top three institutions regarding publication volume, all based in China, and they demonstrate strong cooperative linkages with one another. Due to geographical proximity, Sun Yat-sen University exhibits particularly close associations with Guangzhou Medical University, Southern Medical University, the University of Hong Kong, and Fujian Medical University, indicating a robust regional collaboration in sorafenib resistance studies. Other institutions also show varying degrees of collaboration, emphasizing the essential role of scientific cooperation in advancing this field. Interestingly, all ten institutions with the highest publication counts are situated in China (as detailed in Table 3). However, when examining average citation frequency per publication, only five institutions, namely, Fudan University, the University of Hong Kong, Shanghai Jiao Tong University, and Nanjing Medical University, surpass the average citation level. This finding suggests that, while Chinese institutions lead in publication volume, their global impact still necessitates further enhancement.

**TABLE 3:** Top 10 institutions ranked by the number of publications.

Rank	Organization	Documents	Citations	Average citations	Total link strength
1	Zhejiang University	71	2,976	41.92	616
2	Sun Yat-sen University	68	1,597	23.49	363
3	Fudan University	59	3,911	66.29	580
4	Nanjing Medical University	40	2,015	50.38	509
5	Southern Medical University	36	1,161	32.25	259
6	Second Military Medical University	35	2,232	63.77	457
7	Shanghai Jiao Tong University	35	1,870	53.43	399
8	The University of Hong Kong	34	2,239	65.85	365
9	Huazhong University of Science and Technology	33	870	26.36	255
10	Shandong University	31	858	27.68	404

Applying Price’s Law to assess author output, we identified 14 authors with the highest publication counts as core authors in this research area, establishing a publication threshold of four papers. Following a thorough quantitative screening, we confirmed a total of 282 core authors (Figure 6B). Notable contributors include Ma, Stephanie from the University of Hong Kong, Professor Cai, Xiujun from Zhejiang University, Professor Tang, Xiaolong from Anhui University of Science and Technology, Professor Zhai, Bo from Harbin Medical University, and Professor Zhang, Wei from Fudan University, who occupy the top five positions in publication volume.

In terms of average citation frequencies, half of the top ten authors exceed the average citation count, suggesting that these influential authors could amplify China’s presence on the global stage, thus enhancing scientific output and overall impact (Table 4). Figure 6B effectively illustrates the collaboration network among core authors, revealing that high-output authors are not isolated; rather, they form colorful clusters, indicating stable collaborative relationships. This analysis not only identifies key institutions and authors in sorafenib resistance research but also underscores the vital importance of collaboration in promoting scientific advancement.

**TABLE 4:** Top 10 authors ranked by the number of publications.

Rank	Author	Documents	Citations	Average citation	Total link strength
1	Ma, Stephanie	14	772	55.14	27
2	Cai, Xiujun	12	1,147	95.58	90
3	Tang, Xiaolong	12	262	21.83	17
4	Zhai, Bo	12	1,066	88.83	70
5	Zhang, Wei	12	496	41.33	5
6	Chen, Wei	11	235	21.36	9
7	Sun, Xueying	11	977	88.82	65
8	Wang, Tao	11	366	33.27	19
9	Ding, Jin	10	696	69.60	34
10	Li, Hong	10	294	29.40	14
11	Li, Jun	10	218	21.80	13
12	Xu, Junjie	10	907	90.70	81

### Research knowledge base

**FIGURE 7:**
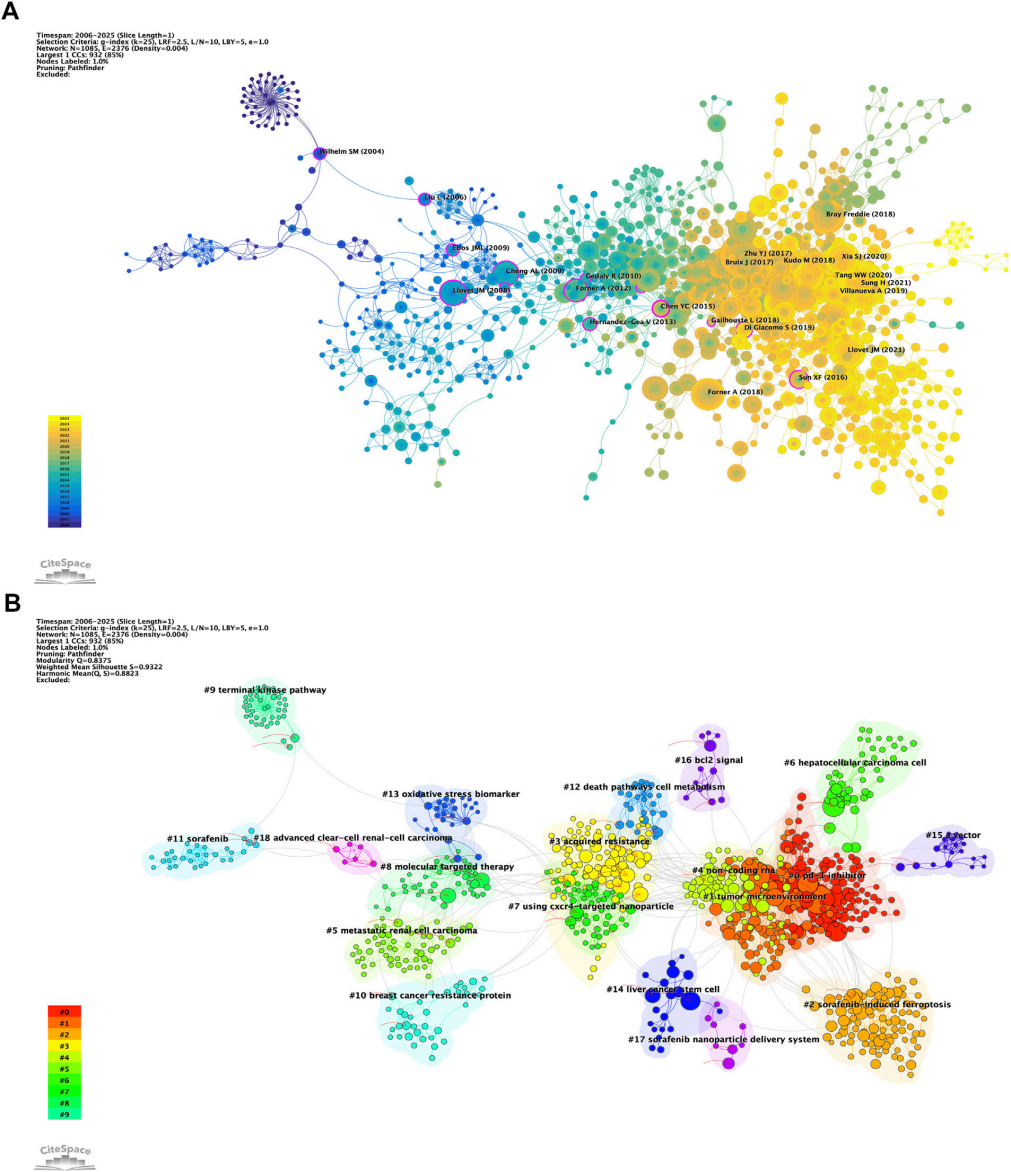
Co-citation network visualization of literature. **(A)** Analysis of co-citation among references. **(B)** Clustering analysis of reference networks.

Co-citation relationships serve as a mapping mechanism among references, employing these citations as nodes to illustrate the knowledge foundation pertinent to a specific subject area. Figure 7A depicts the co-occurrence mapping in the context of sorafenib resistance research within oncology. Larger nodes signify higher co-citation frequencies, which implies a more significant interconnectivity among the cited works. The top ten co-cited articles are distinctly highlighted, and we have also identified the top ten articles based on mediating centrality, as represented by the nodes enclosed in purple circles. The prominence and widespread recognition of these literary works render them invaluable references for researchers within this field, while simultaneously providing fresh perspectives and inspiration for future research trajectories.

In a complementary manner, Figure 7B presents the findings of a co-citation cluster analysis that seeks to uncover the foundational knowledge structure and to define the research boundaries within this domain. The clustering outcomes yield a Q statistic of 0.8375, significantly surpassing the benchmark of 0.3, together with an average weight score, S, of 0.9322, which also exceeds the criterion of 0.7. Collectively, these metrics suggest a homogeneous cluster distribution, asserting that the clustering structure is both sound and dependable. This analysis further affirms the clarity of research boundaries within the sphere of sorafenib resistance, emphasizing the diversity present in research themes.

Ultimately, the analysis delineates 19 distinct cluster labels, each associated with thematic color blocks representing different temporal slices. Among the notable emergent research topics are #17 sorafenib nanoparticle delivery system, #4 non-coding RNA, #1 tumor microenvironment, and #2 sorafenib-induced ferroptosis. These themes not only reflect the dynamic evolution of research in oncology but also underscore the interdisciplinary character of studies focused on sorafenib resistance.

### Research thematic progress

**FIGURE 8:**
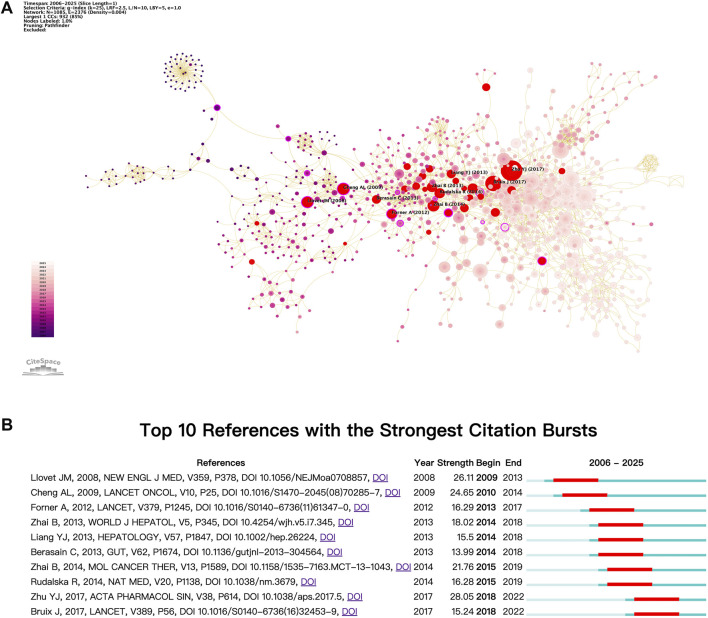
Analysis of burst citation dynamics. **(A)** Co-citation burst detection overview. **(B)** Leading 10 references with highest citation bursts.

In the field of cancer research, burst citation analysis serves as an effective tool for examining significant developments and transformations within specific domains. These foundational papers often exhibit innovative and forward-looking characteristics, thereby enhancing our understanding of the dynamic nature of the field. Figure 8 illustrates the burst citation mapping for sorafenib resistance in cancer research, with red nodes indicating burst citations (Figure 8A). The top ten papers by burst intensity are further detailed in Figure 8B.

Prominent among these publications are studies by Llovet et al. (2008) and Cheng et al. (2009), which conducted pivotal Phase III clinical trials demonstrating the substantial efficacy of sorafenib in treating advanced HCC (Llovet et al., 2008; Cheng et al., 2009). These trials, executed across international multicenter and Asia-Pacific populations, provided compelling evidence that sorafenib significantly prolongs overall survival and time to disease progression compared to placebo. These seminal works established sorafenib as a standard treatment for advanced HCC and laid the groundwork for subsequent investigations into resistance mechanisms and biomarkers. They also revealed variations in patient responses across different regions, thus offering critical insights for personalized treatment strategies. Forner (2012) systematically evaluated sorafenib’s role in advanced HCC, confirming its survival benefit and target mechanisms while addressing toxicity challenges, thus shaping subsequent therapeutic paradigms (Forner et al., 2012). Zhai (2013) conducted the first systematic review summarizing the primary mechanisms of sorafenib resistance, proposing potential solutions and offering a theoretical foundation for subsequent research (Zhai and Sun, 2013). This was followed by an important commentary by Berasain in the journal Gut, which sparked increased interest in the exploration of sorafenib resistance mechanisms among researchers (Berasain, 2013). In a notable study, Liang et al. (2013) approached the issue from the perspective of the tumor microenvironment, particularly focusing on the role of hypoxia. They revealed that continued sorafenib exposure induces a hypoxic microenvironment that activates HIF-1α and NF-κB pathways, leading to the development of resistance in HCC (Liang et al., 2013). Concurrently, Zhai B’s research group (2014) further elucidated the critical role of Akt activation in sorafenib resistance, demonstrating how inhibiting Akt can transform autophagy from a protective to a pro-apoptotic mechanism, thus reversing resistance and presenting a novel targeted therapy strategy for patients with advanced HCC (Zhai et al., 2014). Subsequently, Rudalska et al. employed *in vivo* RNAi screening in mouse models of HCC to identify that the activation of Mapk14 promotes resistance through the Mek-Erk and Atf2 signaling pathways (Rudalska et al., 2014). These studies also indicated that the combination of inhibitors targeting multiple pathways might offer a promising approach to combat sorafenib resistance. Zhu et al. updated the findings on sorafenib resistance mechanisms, providing new direction for future investigations (Zhu et al., 2017). In a significant departure from previous studies, Bruix et al. published a key Phase III clinical trial (RESORCE), which introduced regorafenib as a systemic treatment for patients with HCC who experienced disease progression during sorafenib therapy (Bruix et al., 2017). This study not only offers hope for patients exhibiting sorafenib resistance but also establishes a foundation for future research exploring the combination of regorafenib with other systemic agents.

Overall, the burst cited literature centers on the mechanisms of sorafenib resistance in HCC, highlighting the crucial role of sorafenib in this therapeutic landscape and demonstrating the widespread attention it commands within the research community. These contributions provide a robust foundation for both clinical applications and further scientific inquiry.

### Investigation of keywords and trend topics

**FIGURE 9:**
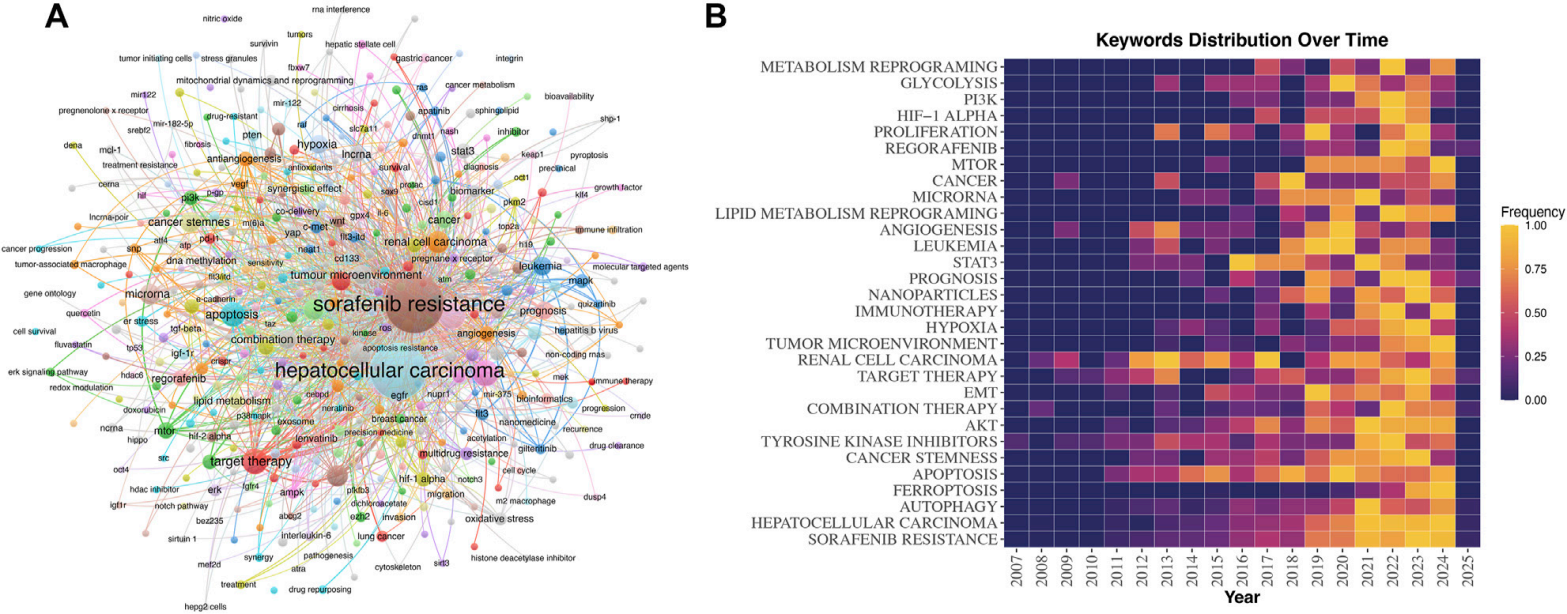
Hot topic network analysis of keywords. **(A)** Keywords network from VOSviewer. **(B)** Time-dependent evolution mapping of keywords.

Keywords function as incisive encapsulations of research themes, effectively summarizing the core insights drawn from the literature. Analyzing high-frequency keywords permits a nuanced visualization of emerging trends within specific scientific fields. In this study, we employed VOSviewer software to dissect the co-occurrence network of terms associated with sorafenib resistance. As illustrated in Figure 9A, the analysis revealed a network comprising 435 keywords with a frequency exceeding two occurrences, with “sorafenib resistance” leading as the predominant term, closely followed by “hepatocellular carcinoma”. This suggests that the exploration of resistance mechanisms is most profound within the context of HCC.

The investigation into these high-frequency keywords encompasses five pivotal domains: disease entities, phenotypes, molecular pathways, signaling cascades, and methodological innovations. Beyond HCC, the analysis also highlights conditions such as RCC, leukemia, DTC, prostate cancer, ovarian cancer, breast cancer, lung cancer, and gastric cancer. This ongoing research underscores the formidable challenges inherent in treating aggressive tumors, necessitating a vigorous pursuit of potential therapeutic avenues.

Prominent phenotypic research foci include ferroptosis, autophagy, apoptosis, tumor stemness, hypoxia, and the tumor microenvironment—hallmarks of malignancy that have garnered considerable attention in the context of sorafenib resistance. Furthermore, key molecular pathways under investigation comprise epithelial-mesenchymal transition (EMT), AKT, mTOR, PI3K, and Stat3 pathways, which represent critical nodes in the regulatory networks of cancer biology. Recent technological advancements, such as non-coding RNAs, nanomaterial delivery systems, and sophisticated bioinformatics tools, are increasingly leveraged to elucidate the complexities of sorafenib resistance and to facilitate translational research aimed at overcoming this challenge, ultimately enriching the therapeutic landscape for cancer patients.

Figure 9B presents a temporal visualization of the evolution of the top 30 high-frequency keywords. The data indicate a period of relatively slow progress from 2006 to 2012, succeeded by a substantial escalation in research activity over the past 5 years, with notable breakthroughs in metabolic reprogramming, lipid metabolism, and ferroptosis. This dynamic shift is intricately linked to the foundational work amassed in preceding years as well as advancements in scientific methodologies. Together, these findings not only illuminate the temporal evolution of key themes within sorafenib resistance research but also highlight the remarkable diversity and complexity characterizing this rapidly advancing domain.

## Discussion

### Temporal distribution and intertumoral heterogeneity

Over the past 2 decades, research output on sorafenib resistance has demonstrated distinct phase-evolution characteristics, with its trajectory closely synchronized with indications expansion and technological advancements. The 2007 approval for HCC triggered a rapid surge in annual publications, achieving a compound annual growth rate of 116.69% and surpassing 40 publications per year by 2013 (Llovet et al., 2008). Following the 2013 approval for DTC (E et al., 2019), the research paradigm underwent a technology-driven transformation. The maturation of single-cell sequencing and organoid modeling technologies enabled mechanistic dissection of resistance to progress from tissue-level analysis to dynamic single-cell regulatory networks, driving annual publication volume beyond 100 articles (Guan et al., 2021; Xian et al., 2022; Zhang et al., 2022). From 2018 to 2024, publications maintained a robust of 15.31%, reflecting the synergistic interplay between precision oncology demands and scientific innovation capabilities (Chen et al., 2019; Xiang et al., 2024).

Analysis of cancer-type distribution reveals marked heterogeneity in sorafenib resistance research. HCC dominates the research landscape with an overwhelming 79.48% share, while RCC (4.58%) and TC (0.99%) remain underrepresented. This disparity stems from distinct molecular pathobiology and clinical challenges across malignancies: Research on sorafenib resistance in HCC primarily addresses the significant global burden of the disease and the high rates of drug resistance observed. Additionally, hyperactivation of angiogenic pathways and remodeling of the tumor microenvironment (TME) are considered crucial mechanisms that may contribute to sorafenib resistance in HCC (Zhang et al., 2022; Elms et al., 2022). For RCC, molecular subtype heterogeneity underlies differential resistance patterns, with HIF2-VEGF axis regulatory divergence emerging as a pivotal mechanism explaining therapeutic response stratification (Zhao et al., 2015; Choueiri and Kaelin, 2020). In DTC, concurrent suppression of MAPK and AKT/mTOR signaling pathways, paradoxically coupled with compensatory protective autophagy, has been established as the key adaptive mechanism underlying resistance in radioiodine-refractory subtypes (Holm et al., 2022; Yi et al., 2018; Liu et al., 2016).

Notably, the research scope has expanded beyond solid tumors to hematological malignancies. Despite lacking formal approval for myeloid leukemia, sorafenib’s off-label use in FLT3-ITD mutated acute myeloid leukemia (AML) and demonstrated clinical activity in FLT3-wildtype AML have contributed to 2.89% of total publications in this domain (Borthakur et al., 2011; Roskoski, 2020; Pratz et al., 2010). This phenomenon mirrors the extreme heterogeneity inherent to hematologic cancers—while FLT3 inhibitor combination strategies significantly improve survival in mutated patients, they concurrently drive resistance evolution through mechanisms such as kinase domain D835 mutations or RAS/MAPK bypass activation, providing unique models for mechanistic studies (Antar et al., 2020; Smith et al., 2015).

### Global research landscape and collaborative networks

The global research landscape in sorafenib resistance is characterized by distinct geographic and institutional collaboration patterns. China and the United States dominate this field, driven by substantial investments in national cancer initiatives and precision medicine programs. Within the European Union, countries such as Italy, Germany, Spain, and France demonstrate robust research output and international cooperation, likely attributable to policy prioritization of aging-associated malignancies like HCC and the European Organization for Research and Treatment of Cancer (EORTC)-facilitated clinical trial integration. These collaborative frameworks, aligned with disease burden and funding synergies, underscore the critical role of multinational networks in addressing complex therapeutic challenges (Siegel et al., 2024; Bray et al., 2024).

At the institutional level, Zhejiang University emerges as a global leader through multidimensional innovations. Mechanistically, its researchers have systematically deciphered key resistance drivers, including HIF/YAP signaling axis activation, non-coding RNA regulatory networks (e.g., lncRNA-POIR/miR-182-5P crosstalk), and ferroptosis evasion via NRF2/SLC7A11 dysregulation (Zhou et al., 2016; Chen et al., 2021; Ren et al., 2021; Li et al., 2022). Therapeutically, breakthroughs encompass a self-assembled nanocarrier system with >85% drug-loading efficiency (Zhang et al., 2025), CRISPR-based genome-wide screening for novel target identification (e.g., SGOL1) (Sun et al., 2018), and combinatorial epigenetic/immune therapies that significantly enhance resistance reversal (Ma et al., 2021; Chen et al., 2016). Clinically, the integration of multimodal biomarkers (e.g., aerobic glycolysis indices, phospho-ERK activity) with multi-omics deep learning models and spatial transcriptomics has enabled resistance stratification, bridging mechanistic discovery to clinical validation, with several therapies advancing to trials (Chen et al., 2016; Pan et al., 2021).

Research conducted at the University of Hong Kong, led by Professor Stephanie Ma, has established a paradigm-shifting framework focusing on tumor microenvironmental stressors (e.g., hypoxia, metabolic reprogramming) that drive HCC resistance (Ma et al., 2017; Che et al., 2021). This work elucidates the interplay between epigenetic regulation, cancer stemness maintenance, and metabolic adaptation, offering a closed-loop translational model from mechanistic exploration to therapeutic innovation (Wong et al., 2020; Cheng et al., 2018; Mok et al., 2022; Gao et al., 2023). By identifying actionable targets within stress-responsive signaling networks, these contributions exemplify the convergence of precision medicine and interdisciplinary collaboration in overcoming sorafenib resistance, further solidifying the global momentum toward HCC treatment optimization.

### Knowledge framework and thematic advancements

The intellectual architecture of sorafenib resistance research has undergone systematic evolution, as revealed by co-citation network analysis and burst detection. Initial investigations centered on RAF/MEK/ERK signaling deconstruction, with Wilhelm et al.’s seminal characterization of sorafenib (BAY 43-9006) as a dual-action multi-kinase inhibitor (RAF/VEGFR/PDGFR) establishing the #9 terminal kinase pathway cluster as the foundational paradigm (Wilhelm et al., 2004). Subsequent validation in HCC models via MEK/ERK phosphorylation blockade expanded therapeutic validation into the #11 sorafenib cluster (Liu et al., 2006), while the SHARP trial extended this paradigm to pan-cancer VEGF/PDGF inhibition through the #18 advanced clear-cell renal-cell carcinoma cluster (Llovet et al., 2008; Cheng et al., 2009; Ebos et al., 2009). Despite these advances, early 2D/xenograft models inadequately addressed resistance heterogeneity, a limitation partially mitigated by the 2010 identification of Ras/Raf-PI3K/AKT/mTOR crosstalk in PI-103 combination studies—a precursor to modern resistance network modeling (Gedaly et al., 2010).

A paradigm shift occurred with TME dynamics elucidation (Hernandez-Gea et al., 2013). Chen et al. demonstrated hypoxia-induced CXCR4-mediated immunosuppression under sorafenib pressure, propelling the #1 tumor microenvironment cluster and transitioning resistance mechanisms from tumor-autonomous to niche co-evolution models (Chen et al., 2015). This reorientation explained the clinical insufficiency of RAF monotherapy (as per the #9 terminal kinase pathway cluster) and catalyzed emergence of the #0 PD-1 inhibitor cluster, wherein PD-1 blockade efficacy strictly required CXCR4 co-inhibition—highlighting microenvironmental reprogramming as a therapeutic imperative. Concurrently, cell death regulation studies revealed MT-1G-mediated ferroptosis suppression via Nrf2 antioxidant activation (#2 sorafenib-induced ferroptosis cluster), complementing oxidative stress biomarker profiling in the #13 oxidative stress biomarker cluster (Sun et al., 2016). Epigenetic mechanisms entered the resistance network through DNMT1 inhibition studies (Differentiation Therapy by Epigenetic Reconditioning, 2018), which restored differentiation phenotypes in resistant HCC stem cells, thereby bridging genomic and functional resistance determinants (Gailhouste et al., 2018).

Systems biology has since driven three transformative advances: therapeutic hierarchy standardization, mechanism-guided combination design, and dynamic intervention precision. VEGFR/PDGFR axis modulation established second-line standards, while molecularly stratified immune combinations demonstrated tumor niche reprogramming efficacy (Bruix et al., 2017; Kudo et al., 2018; Llovet et al., 2021). Microenvironment-epigenetic dual targeting (PD-1/CXCR4 synergy and non-coding RNA pathway reversal) overcame empirical combination limitations through mechanistic integration (Chen et al., 2015). Resistance heterogeneity was addressed via systems-derived subpopulation strategies—NRF2 inhibition for ferroptosis-resistant clusters and Wnt targeting for stemness-dominant subtypes (Ren et al., 2021; Huang et al., 2017). Crucially, dynamic monitoring systems integrating oxidative stress signatures, drug transporter activity, and epigenetic biomarkers enable real-time resistance surveillance, transitioning clinical decision-making from static classification to adaptive therapeutic iteration (Yuan et al., 2021; Fang et al., 2024; Li et al., 2023b). This paradigm evolution from empirical management to mechanism-guided precision underscores systems biology’s indispensable role in translational bridging, where multi-omics integration (genome-epigenome-metabolome-transcriptome) and computational modeling synergize to decode resistance complexity.

### Hotspots and research frontiers

Through systematic keyword analysis, this study elucidates research hotspots, evolutionary trends, and mechanistic insights into sorafenib resistance in oncology. HCC represents the primary focus of sorafenib resistance research, with well-defined mechanisms spanning pharmacokinetic and molecular dimensions (Sonbol et al., 2020; Abdel-Rahman et al., 2013). Key resistance drivers include dysregulated drug transport (e.g., ATP binding box transporters and exosomes), upregulating metabolic clearance, and reactivation of RTK and downstream effectors (Ras/Raf/MEK/ERK, VEGFR and SHP2) (Zhu et al., 2017; Tang et al., 2020; Jiang and Dai, 2023; Cheng et al., 2020). Besides, hypoxic tumor microenvironments stabilize HIF-1α/2α to upregulate drug efflux pumps (ABCB1/MRP1), while metabolic reprogramming through enhanced glutaminolysis and lipid metabolism supports redox homeostasis (Huang et al., 2022; Jiang et al., 2025; Mendez-Blanco et al., 2018). Recently, mounting evidence highlights the association between sorafenib resistance in HCC and epigenetic regulatory mechanisms—including DNA methylation, histone modifications, and dysregulation of non-coding RNAs—as well as regulated cell death (RCD) pathways, particularly the critical roles of autophagy and ferroptosis in mediating therapeutic resistance to sorafenib. Meanwhile, the compensatory activation of signaling pathways and their crosstalk constitute a pivotal mechanism underlying sorafenib resistance. Notably, the PI3K/AKT pathway and the JAK/STAT3 pathway can abrogate the cytotoxicity of sorafenib by eliciting apoptosis resistance via SHP-1-mediated signaling (Mor et al., 2005; Chen et al., 2012). These advancements, as detailed in the seminal review by W. Tang and Z. Jiang, highlight the critical need for mechanism-driven combination therapies to overcome sorafenib resistance in HCC (Tang et al., 2020; Jiang and Dai, 2023).

RCC demonstrates overlapping yet distinct resistance mechanisms compared to HCC (He et al., 2021). PI3K/Akt/mTOR hyperactivation emerges as a central resistance driver, with preclinical evidence showing enhanced sorafenib efficacy when combined with pathway inhibitors (Tei et al., 2015). Hypoxia adaptation via HIF stabilization paradoxically limits sorafenib’s anti-angiogenic effects through VEGFR inhibition-induced compensatory HIF-2α/COX2 upregulation (Zhao et al., 2015). Beclin-1-mediated autophagy activation and miR-30a dysregulation complicate sorafenib resistance in RCC, necessitating combination approaches with autophagy inhibitors and miR-30a-based therapies (Zheng et al., 2015).

In addition to these mechanisms, the p53-mediated apoptotic pathway plays a significant role in sorafenib resistance in RCC (Bao et al., 2018). The upregulation of ANGPTL3 enhances sorafenib sensitivity, while increased GSK-3 activity may contribute to acquired resistance (Bao et al., 2018; Kawazoe et al., 2012). Collectively, these elements underscore the intricate interplay of resistance mechanisms in RCC and illuminate prospective therapeutic avenues for their mitigation.

In myeloid leukemia, although the clinical data of sorafenib are encouraging, the issue of resistance remains a significant challenge (Borthakur et al., 2011; Roskoski, 2020; Pratz et al., 2010; Wu et al., 2018). The mechanisms of resistance primarily involve intrinsic insensitivity of FLT3 mutations to specific FLT3 inhibitors or the presence of activated alternative survival pathways (Antar et al., 2020). Acquired resistance mechanisms include clonal evolution, such as loss of FLT3 clones, acquisition of resistance mutations, autocrine FLT3 signaling, FLT3 overexpression, protection by the bone marrow microenvironment, increased autophagy, and activation of downstream or bypass signaling pathways (Joshi et al., 2021).

While only HCC, RCC, and DTC have been sanctioned for sorafenib treatment, preclinical explorations at cellular and animal model levels continue for other malignancies, such as lung cancer and prostate cancer (Mu et al., 2021; Karkampouna et al., 2021). Mechanistically, sorafenib resistance encompass molecular heterogeneity at the tumor cell level, overactivation and crosstalk of signaling pathways, genomic instability related to drug transport and metabolism, and the complexity of the tumor microenvironment. Additionally, metabolic reprogramming and resistance to cell death pathways that evolve during the process of resistance acquisition represent critical adaptive alterations contributing to sorafenib resistance. In reality, drug resistance mechanisms are interconnected rather than being isolated entities, especially within the framework of sorafenib-based TKI resistance pathways. Therefore, investigating sorafenib resistance is essential for gaining insights into the resistance mechanisms of other medications, including lenvatinib, regorafenib, and various kinase inhibitors (Jiang et al., 2025).

In clinical practice, the epigenetic inhibitor targeting HDAC4, Tasquinimod, has not demonstrated clinical benefits as a standalone treatment for HCC (Escudier et al., 2017). However, when combined with sorafenib, it can inhibit the formation of the protein complex between HDAC4 and the transcription factor MEF2D, which in turn suppresses the activation of the downstream SPRY4-dependent ERK signaling pathway and reverses sorafenib resistance in preclinical models (Ma et al., 2021). Furthermore, the aberrantly high expression of the MEF2D gene in HCC patients may serve as a significant biomarker for predicting their response or resistance to sorafenib treatment. In line with this, an early-phase clinical trial initiated by the Massey Cancer Center, which is investigating the combination of sorafenib and Vorinostat, has also provided preliminary evidence of the potential of HDAC inhibitors in the treatment of HCC (Gordon et al., 2019). Recent advancements in omics technologies, particularly the emergence of single-cell resolution sequencing, have enabled the integration of multi-omics data and the application of machine learning algorithms (Yu and Ma, 2024; Zhang et al., 2024). These developments have brought new biomarkers, such as PD-1, to the forefront, along with analyses of the immune microenvironment characterized by tumor-infiltrating lymphocytes (TILs), which are becoming significant research areas (Zhang et al., 2023; Chen et al., 2024). To improve treatment efficacy, personalized strategies are being developed based on biomarker profiles that include traditional markers like AFP, GPC3, and DCP, as well as newer indicators such as PD-L1 and genetic mutations (e.g., CTNNB1, TERT, TP53) (Yu and Ma, 2024). This approach offers a range of options, including targeted therapies, immune checkpoint inhibitors, and combination treatments (Chen et al., 2016; Chen et al., 2015). Regular biomarker testing is crucial for early detection and ongoing assessment, informing clinical decisions on therapy continuation or adjustments. Thus, biomarker discovery and monitoring are essential for effective clinical trial design and research translation into practice.

Future research should focus on several key areas: First, harnessing multidisciplinary integration—encompassing big data analysis, molecular biology, cellular and animal models, as well as pharmacokinetics and pharmacodynamics—to synthesize multimodal data and better understand the mechanisms underlying resistance. Second, efforts should be directed towards identifying and validating molecular biomarkers for patient stratification in personalized therapy. Third, a deeper exploration of the molecular biology of cancer initiation is essential for uncovering alternative therapeutic and prognostic targets, thereby improving treatment efficacy. Finally, it is important to expedite the translation of clinical trials to investigate combination targeted therapies and accelerate the development of new drugs.

This study’s bibliometric analysis of sorafenib resistance has certain inherent limitations. Our reliance on the WoS database may introduce biases due to the lack of data from other databases such as PubMed, Google Scholar, Scopus, and EMBASE. Additionally, by primarily including English-language publications, we may overlook valuable insights from non-English sources. Furthermore, citation inflation could distort the perceived impact of certain studies, leading to an uneven representation of key topics in the field. Recognizing these biases is crucial for contextualizing our findings, and future research would benefit from a broader approach that incorporates diverse databases and languages to provide a more comprehensive view of the literature.

## Conclusion

In conclusion, this study elucidates the current research landscape of sorafenib resistance, revealing critical trends and gaps. Since 2006, research on sorafenib resistance has shown continuous growth, with China and the United States leading in publication volume and research impact. Studies are particularly advanced in the fields of HCC, RCC, DTC, and myeloid-related leukemias. Key mechanisms explored include drug transport and clearance, metabolic reprogramming, programmed cell death, interactions within the tumor microenvironment, and epigenetic regulatory mechanisms. With advancements in omics and computational biology, the era of big data necessitates the integration of diverse research findings to enhance our understanding of resistance mechanisms and foster the development of novel therapies. Continued collaboration and exploration are vital to advance our understanding of this complex challenge in cancer therapy.

## Data Availability

The original contributions presented in the study are included in the article/supplementary material, further inquiries can be directed to the corresponding author.
